# Urinary microRNAs and Their Significance in Prostate Cancer Diagnosis: A 5-Year Update

**DOI:** 10.3390/cancers14133157

**Published:** 2022-06-28

**Authors:** Jaroslav Juracek, Marie Madrzyk, Michal Stanik, Ondrej Slaby

**Affiliations:** 1 Central European Institute of Technology, Masaryk University, 625 00 Brno, Czech Republic; jaroslav.juracek@ceitec.muni.cz (J.J.); marie.madrzyk@ceitec.muni.cz (M.M.); 2 Department of Urologic Oncology, Clinic of Surgical Oncology, Masaryk Memorial Cancer Institute, 656 53 Brno, Czech Republic; stanik@mou.cz; 3 Department of Biology, Faculty of Medicine, Masaryk University, 625 00 Brno, Czech Republic

**Keywords:** prostate cancer, microRNA, urine, extracellular vesicles, diagnosis

## Abstract

**Simple Summary:**

Current diagnostics of prostate cancer often show unsatisfactory results, leading to delayed detection or overtreatment. Urinary microRNAs are a class of promising non-invasive biomarkers. Although many studies have been conducted on this topic in the last five years, there is little agreement on the data obtained. This review aims to discuss new knowledge but also focuses on technical aspects affecting urinary miRNA analysis.

**Abstract:**

Current routine screening methods for the diagnosis of prostate cancer (PCa) have significantly increased early detection of the disease but often show unsatisfactory analytical parameters. A class of promising markers represents urinary microRNAs (miRNAs). In the last five years, there has been an extensive increase in the number of studies on this topic. Thus, this review aims to update knowledge and point out technical aspects affecting urinary miRNA analysis. The review of relevant literature was carried out by searching the PubMed database for the keywords: microRNA, miRNA, urine, urinary, prostate cancer, and diagnosis. Papers discussed in this review were retrieved using PubMed, and the search strategy was as follows: (urine OR urinary) WITH (microRNA OR miRNA) AND prostate cancer. The search was limited to the last 5 years, January 2017 to December 2021. Based on the defined search strategy, 31 original publications corresponding to the research topic were identified, read and reviewed to present the latest findings and to assess possible translation of urinary miRNAs into clinical practice. Reviews or older publications were read and cited if they valuably extended the context and contributed to a better understanding. Urinary miRNAs are potentially valuable markers for the diagnosis of prostate cancer. Despite promising results, there is still a need for independent validation of exploratory data, which follows a strict widely accepted methodology taking into account the shortcomings and factors influencing the analysis.

## 1. Introduction

Prostate cancer (PCa) is estimated to be the second most often diagnosed cancer globally and the fifth most common cause of cancer-related death in men [1]. It is typically a slow-growing tumor, 40% of men showing no clinical symptoms at the time of diagnosis. More than 99% of patients have a survival rate of at least five years in the case of localized disease; however, the rate drops to 40–70% if they are diagnosed at the advanced or metastatic stage [2,3]. The risk of clinically significant prostate cancer is related to age, ethnicity, family history, prostate-specific antigen (PSA) level, free/total PSA ratio, and findings on digital rectal examination (DRE). These clinical parameters are important for PCa diagnosis, with DRE being the main method of clinical T stage evaluation [4]. Recently, multiparametric magnetic resonance imaging of the prostate showed good sensitivity for the detection of clinically significant cancers [5].

The use of PSA as a biomarker for detecting prostate cancer caused a sharp increase in PCa incidence and lowered the number of metastatic or locally advanced tumors diagnosed (the specificity and sensitivity range from 20% to 40% and from 70% to 90%, respectively). However, routine clinical screening also led to overdiagnosis and unnecessary treatment of indolent cancers that do not pose a threat to life or health. Furthermore, PSA is a prostate- but not cancer-specific marker, and it may be elevated in benign prostate diseases such as prostatitis or benign prostatic hyperplasia (BPH) [6]. Adverse effects of overdiagnosis include psychological harm from false-positive test result, pain, bleeding, fever, infection, and urinary problems connected to prostate biopsy. Subsequent cancer treatment can cause erectile dysfunction, urinary incontinence, bowel dysfunction, and possibly premature death [7].

Despite advances in diagnosis implementing, for example, PSA derivatives, circulating tumor cells, cell-free DNA, circulating RNA, proteins, and peptides, there is a lack of biomarkers that would have sufficient sensitivity and specificity and would also be able to distinguish between different subtypes of PCa [8]. Current trends prefer minimally invasive biomarkers, which are easily available and stable in various body fluids and reflect the clinical–pathological characteristics of the disease. One of the most studied groups of such biomarkers is circulating microRNAs (miRNAs). A specific subgroup is represented by urinary miRNAs, the significance of which is evident in genitourinary cancers.

## 2. microRNAs in Prostate Cancer

miRNAs are a class of small non-coding RNAs that regulate gene expression at the post-transcriptional level. They are 20–24 nucleotides long and have an indispensable function in many biological processes such as cell development, differentiation and proliferation, DNA damage repair, apoptosis, and cellular communication [9]. As one miRNA regulates many mRNAs, its dysregulated expression can have a profound effect on cellular homeostasis. Because miRNA expression can be highly tissue specific, disrupted levels are implicated in the molecular pathogenesis of various diseases, including cancer. Here, miRNAs play an important role in tumor initiation and development by acting as tumor-promoting (oncogenic) or tumor-suppressing miRNAs [10].

The first large-scale studies of dysregulated miRNA expression in prostate cancer tissue laid the foundations for a better understanding of miRNA involvement in prostate cancer pathophysiology. Such signatures can differentiate not only between tumor and non-tumor tissue but can also classify PCa according to disease aggressiveness. Most of these miRNAs regulate signaling pathways critical for prostate cancer, such as Wnt/β-catenin signaling [11] (embryogenesis, cell proliferation), AR signaling [12] (proliferation, apoptosis, etc.), NF-κB signaling [13] (inflammation), JAK/STAT signaling [14] (immunity) and receptor tyrosine kinase signaling [15] (cell growth, differentiation, etc.).

A typical example represents miR-21, an oncomir that regulates multiple signaling pathways. miR-21 by itself is a direct transcriptional target of the androgen receptor (AR), but at the same time, it increases AR expression in PCa cell lines [16], potentially by targeting tumor suppressors. miR-21 targets and inhibits the expression of the tumor suppressor gene PTEN to promote prostate cancer cell proliferation and invasion [17]. Furthermore, miR-21 regulates cell invasiveness by directly controlling RECK (reversion-inducing cysteine-rich protein with Kazal motifs), a key inhibitor of several metalloproteinases [18]. Another well-described oncogenic miRNA is miR-210, whose overexpression in metastatic PCa was positively correlated with serum PSA levels, Gleason score, and bone metastasis status. Upregulation of miR-210 leads to sustained activation of NF-κB signaling by targeting its negative regulators TNIP1 (TNF-α Induced Protein 3 Interacting Protein 1) and SOCS1 (Suppressor of Cytokine Signaling 1), which results in epithelial–mesenchymal transition (EMT), invasion, and migration [19].

Tumor-suppressor miRNAs, such as the miR-143/miR-145 cluster, often relate in prostate cancer to biological processes such as EMT, invasiveness, metastasis formation, or therapy response, which are typical of advanced stages of the disease. miR-143/miR-145 can suppress autophagy by downregulating ATG2B and thus sensitizing prostate cancer cells to radiation [20]. Moreover, this cluster downregulates EMT by targeting the zinc-finger E-box binding homeobox 2 (ZEB2), a prototypical EMT activator [21]. Similar functioning was described within members of the miR-200 family, which suppress tumor invasion, metastasis, and chemosensitivity [22,23].

## 3. Urinary miRNAs

With all that said, miRNAs are justly of scientific interest as a class of promising biomarkers in prostate cancer diagnosis and management. In addition to their intracellular roles, miRNAs are secreted by donor cells as a part of intercellular communication that includes regulation of gene expression in recipient cells. Cell-free miRNAs have been detected in different body fluids, including plasma, serum, and urine, and they show remarkable stability in both tissue and these so-called liquid biopsies. It has been proposed that miRNA can be released to the extracellular space by passive leakage from broken cells in cases of tissue injuries, chronic inflammation, etc., or they can be actively secreted by the cells in the form of extracellular vesicles (EVs), as well as bound to high-density lipoproteins.

Compared to blood, the expression profiles of urine miRNAs in PCa have not been extensively studied, and most of the studies concerned focus on miRNA determination in extracellular vesicles, urine sediments, or whole urine [24]. Although urinary EVs were found to contain a significantly higher concentration of miRNAs than cell-free urine [25], their purification requires the use of specialized equipment such as ultracentrifuges, which is not practical for most clinical diagnostic laboratories. While miRNAs found in urine supernatant are cell free and emerge from microvesicles, using urine sediment for miRNA profiling might skew the expression data, as the sediment also contains epithelial cells that have variable proportions to tumor cells among subjects [26].

**Figure 1:**
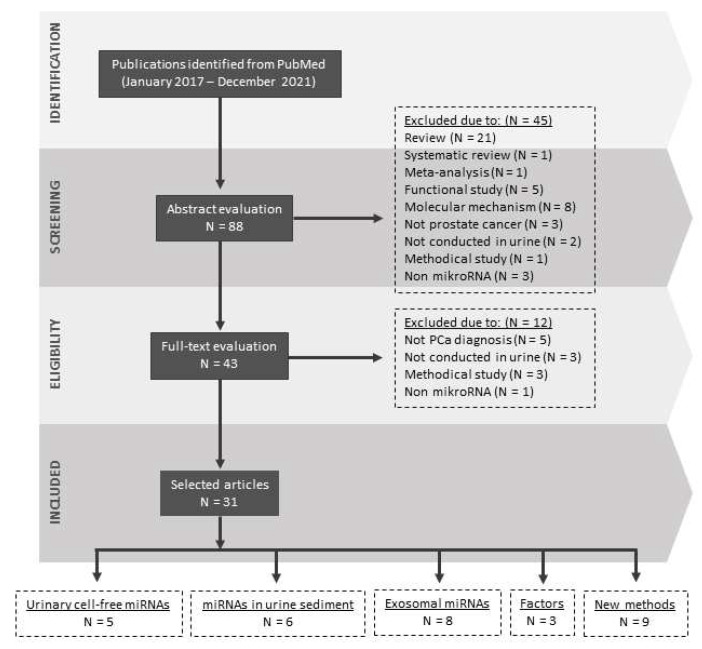
A flowchart illustrating strategy for literature search and relevant studies selection.

The first study reporting changes in miRNA concentration in the urine of prostate cancer patients was published in 2012. Bryant et al. have quantified selected miRNAs, of which miR-107 and miR-574-3p were present at significantly higher concentrations in the urine of men with cancer compared to controls [27]. These observations have led to growing interest and an increasing number of investigations in recent years. Currently, there are a considerable number of studies describing the importance of urinary miRNAs in prostate cancer diagnostics and management [28,29]. However, these studies often suffer from heterogeneity and inconsistency within the miRNA analysis process and thus the published data [30]. Undeniably, it is mainly because there is a lack of recommendations or guidelines for urine sampling and processing as well as for miRNA isolation and quantification [31]. Therefore, this review aims not only to summarize new knowledge in the field, but also to unveil whether these shortcomings were corrected. The study selection is illustrated in Figure 1.

## 4. Urinary miRNAs and Prostate Cancer Diagnostics

Urine represents an easily accessible biological specimen and a source of extracellular biomarkers. Among the transcripts measured in urine as part of the combined panels currently approved for additional PCa diagnostics are the PCA3 and TMPRSS2-ERG tests; however, neither can significantly outperform the PSA test. The potential use of urinary miRNAs in clinical practice is supported not only by their biological characteristics but also by their exceptional stability and good analytical performance. Table 1 refers to miRNAs with diagnostic potential analyzed in cell-free urine or urine sediment.

### 4.1. Urinary Cell-Free miRNAs

When searching for new biomarkers, the most valuable are considered biomarker studies with large-scale screening (discovery phase) followed by one or more levels of validation. Such exploratory investigations provide a good basis for biomarker selection, but at the same time, they are financially and materially demanding. In their study, Byun et al. determined a miR-based urinary signature in urine supernatant from PCa patients using Agilent Human miRNA Microarray. On the subset of 12 miRNAs that showed significant differences between PCa and BPH samples, they used a ratio analysis, since urinary levels of upregulated miRNAs were compared with levels of downregulated miR-3659. For subsequent validation, the urinary miR-1913 to miR-3659 ratio was selected, where it was significantly higher in PCa than in BPH, with even improved diagnostic power within the PSA gray zone (AUC = 0.82) [32].

Over a period of five years, several large-scale interconnected and innovative studies have been published. In the pioneer study by a team from Aarhus, Denmark, novel miRNA-based prostate cancer biomarkers were determined in cell-free urine. Using custom RT-qPCR array, urine miRNAs were profiled within a unique cohort counting more than 200 specimens. Instead of validating only significantly dysregulated miRNAs (14 miRNA upregulated, 30 downregulated), the authors used the same technology also within a new independent cohort. They successfully validated six upregulated miRNAs and 22 downregulated miRNAs. In addition, they created diagnostic ratio models that compared all possible two-miRNA combinations. The diagnostic performance of all miRNA models was statistically evaluated and finally led to the construction of a definitive three-miRNA model (miR-222-3p*miR-24-3p/miR-30c-5p), which allows for distinguishing between PCa and BPH patients with an AUC of 0.95 but was also significantly associated with PC aggressiveness [33]. To increase robustness and to verify clinical benefits, identified diagnostic miRNAs, including the mentioned three-miRNA model, were validated in the follow-up study [34]. Adding two more distinct independent PCa cohorts, the authors validated 29 miRNAs significantly dysregulated in previous research. Moreover, the described miRNA model distinguished PCa from BPH in all cohorts but also predicted prostate biopsy results even for patients within the so-called gray zone (PSA level ≤ 10 ng/mL) [34]. A machine learning-based approach for the selection of diagnostic urine miRNAs was used by Lekchnov et al. in the 2018 paper. After high-capacity miRNA screening using a custom qPCR panel, they normalized the data with the pair ratio method and thus compiled all possible combinations of miRNA pairs. These pairs were compared between patients with PCa, BPH, and healthy donors and significant combinations highlighted by the Random Forest-based algorithm. As a result, two pairs, miR-107-miR-26b-5p and miR-375-3p-miR-26b-5p, demonstrated good diagnostic efficacy when distinguishing PCa patients from healthy donors with an AUC of 0.93 and 0.83, respectively [35]. Identified miRNAs with diagnostic potential were further validated in a follow-up study—this time, a subset of 12 miRNAs was analyzed in clarified urine, urine extracellular vesicles and blood plasma. Again, miRNAs were combined in ratios and compared between the biological materials mentioned. Although several miRNA combinations from urine supernatant were able to distinguish PCa and healthy donors (one pair), PCa and BPH (nine pairs), and BPH and healthy donors (two pairs), most of the significantly differentially expressed miRNA pairs originating in EVs. The highest diagnostic potential was described within the miR-125b/miR-30e, miR-200/miR-30e, miR-205/miR-30e, miR-31/miR-30e, miR-660/miR-30e, and miR-19b/miR-92a ratios, detecting PCa patients with 100% sensitivity and 100% specificity [36].

### 4.2. miRNAs in Urine Sediment

Measurements in urine sediment allow for the detection of significantly higher levels of miRNAs; however, the content can change under pathological conditions. In 2021, Hasanoğlu performed a urine sediment and serum miRNA profile analysis uncovering a set of 49 differentially regulated miRNAs in samples from PCa patients compared to healthy controls. However, only miR-320a was validated as a valuable biomarker in early PCa diagnosis [37]. In a pilot study by Guelfi et al., miRNAs were analyzed in urine exfoliated cells within urine sediment from PCa patients. Using a small RNA sequencing method, they identified 236 miRNAs, which were later subjected to computational functional analysis. As a result, they identified several pathways connected to detected miRNAs, from which specific genes, such as Ras, PTEN, and Cyclin D1, are involved in PCa signaling. To validate the diagnostic potential, members of the let-7 family were selected and analyzed within PCa subjects and age matched healthy controls. The results showed a significant downregulation of all members of the let-7 family in patients with PCa and confirmed that urine exfoliated prostate cells can serve as a promising biological specimen within PCa detection [38].

Some studies focus, due to the large amount of expression data already presented or stored within database repositories, on validating already published results. Investigating the most prevalent urological tumors, Ghorbanmehr et al. evaluated the urinary levels of miR-21-5p, miR-141-3p, and miR-205-5p in patients with bladder (BCa), prostate cancer, benign prostatic hyperplasia, and healthy controls. All selected oncogenic miRNAs appeared to be significantly upregulated in cancer-related samples with higher levels in PCa compared to BCa. Despite that these miRNAs were selected based on bladder cancer profiling studies, they showed good discriminating abilities within PCa diagnostics with the same sensitivity as PSA, but higher specificity [39]. Based on published research in the field, Nayak et al. selected miR-182 and miR-187 as potential diagnostic and prognostic markers of PCa. While in PCa tissues, both miRNAs differed significantly compared to the control, in the urine sediment of the same patients, the difference was not statistically significant, nor did it show a correlation with the clinical–pathological characteristics of PCa [40]. In the investigation by Borkowetz et al., 12 miRNAs previously shown to be dysregulated in PCa were analyzed in urine sediment to confirm their diagnostic capabilities. However, only miR-16 and miR-195 were significantly dysregulated in PCa patients. Conversely, these small non-coding RNAs outperformed serum PSA testing and when combined (individually or simultaneously) with PSAD (PSA density) showed improved diagnostic power (miR-16/PSAD—AUC = 0.834; miR-195/PSAD—AUC = 0.801, miR-16/miR-195/PSAD—AUC = 0.849) [41].

It is the urine fraction used for miRNA analysis in which most of the published studies differ. For this reason, some publications include the concurrent analysis of several types of biological specimens [35,36,42]. Foj et al. evaluated five miRNAs commonly dysregulated within PCa in urinary sediments and urinary exosomes. Even for such a small number of molecules, they observed differences within the miRNA level in the urine fractions used. While miR-21 and miR-375 showed good diagnostic properties in both urine sediment and exosomes, miR-141 was significantly dysregulated in PCa patients compared to healthy controls only when measured in urine sediment. This miRNA was also significantly correlated with the Gleason score (*p* = 0.034). Conversely, let-7c showed insignificant differences between PCa and HC in urine sediment, while in urine exosomes, this miRNA was not only upregulated, but also correlated with clinical stage (*p* = 0.023) Moreover, miR-21, miR-141, and miR-214 in urine sediment and miR-21, miR-375, and let-7c in urinary exosomes were associated with disease aggressiveness when significant deregulation in intermediate/high-risk PCa versus low-risk/healthy subjects was observed [42].

Despite a rather limited number of studies, there is a noticeable overlap between potential diagnostic miRNA. Among studies conducting a large-scale screening, miR-30b, miR-31, miR-24, and miR-125b were described as dysregulated in two studies [33,35] (here, we do not count the follow-up studies by Fredsøe [34] and Konoshenko [36], as they validated previous work). If we also include analyses that have validated already published data, dysregulation of miR-141 was detected between PCa patients and the control group in three studies [33,39,42], while two articles consistently described the alteration of miR-21 [39,42], miR-205 [36,39], miR-375 [35,42], let-7c [38,42] and miR-16 [35,41].

### 4.3. Exosomal miRNAs

When comparing more urine fractions, EVs were often the source of a larger quantity of miRNAs [35,43]. This is in line with the latest trend shifting the focus to exosomes, microvesicles, and other extracellular vesicles (EVs) as a potential reservoir of clinically relevant biomarkers for disease detection, progression, and treatment response. The main advantage lies in protecting their content from degradation. Most EVs are roughly 30 to 1000 nm in diameter, and depending on their biogenesis, they can be categorized into several subgroups. While microvesicles (ectosomes) are formed by outward budding and fission of the plasma membrane, exosomes are part of the multivesicular bodies that are released to the cell exterior upon their fusion with the plasma membrane [44,45]. The characteristic lipid bilayer of EVs serves as protection against enzymatic digestion of internal components and contributes to their stability [46]. Cells actively release EVs to their exterior and body fluids to mediate cell-to-cell communication with adjacent or distant sites [45,47]. The content of EVs can reflect the biological properties of parental cells, and vesicles derived from cancer cells have been shown to contain tumor-specific molecules such as nucleic acids (NA) and proteins [48].

In the past five years, a considerable number of studies has focused on the detection of urinary miRNAs enveloped in extracellular vesicles as a potential class of non-invasive biomarkers for prostate cancer detection. Generally, the reviewed studies here differ in the method of EV isolation from urinary samples and in the evaluation of the quantity, size, and distribution of the purified vesicles. As it is important for the downstream analysis and assessment of urinary EVs (uEVs), cell-depleted urine samples (urine supernatant) are used in the following studies. The isolation of miRNAs is usually performed using commercial isolation kits. Most analyses are based on RT-qPCR experiments that quantify selected miRNA targets in the exploratory cohort. Fewer studies employ high-throughput technologies, such as NGS or microarray profiling, to identify a higher number of miRNAs. In some studies, detected miRNAs are verified during the validation phase, which involves a larger set of patients (see Table 2).

Using hydrostatic filtration dialysis to isolate EVs, Xu et al. focused on qPCR analysis of the selected miRNAs. Of the four miRNAs measured in the exosomal fraction of the urine supernatant, miR-145-5p and miR-1290 were significantly increased in the group of 60 PCa patients in comparison to the group of 37 BPH patients. The study also demonstrated an increased diagnostic potential of miR-145-5p in combination with the PSA value (AUC = 0.863). Quantification of the other two targets, miR-572 and miR-141-5p, revealed significant differences in exosomal concentration between PCa patients and healthy controls, but not between the BPH and PCa groups [49]. A new automated method for isolation of EVs based on acoustic trapping was presented by Ku et al. During the isolation process, vesicles are exposed to ultrasound, which leads to their aggregation and retention against fluid flow within a microfluidic system. An EV enrichment provided a sufficient amount of RNA for NGS analysis, resulting in the detection of six miRNAs whose level distinguished patients with high-grade PCa (ISUP grade group 4 or higher) and patients with ISUP grade group 3 or lower, including those with no evidence of prostate cancer at biopsy. The differentially expressed miRNAs were later analyzed in the TCGA prostate dataset where miR-10a and miR-27a were significantly upregulated while miR-1 and miR-23b were downregulated in the high-grade group of PCa patients [50].

Another study explored dysregulated miRNA levels in a moderately large cohort of 80 urine samples. In this study, Danarto et al. showed significant differences in miR-21-5p levels between the groups of metastatic and non-metastatic PCa and BPH patients. Statistically significant was also the downregulation of miR-200c-3p in both PCa groups versus the BPH cohort [51]. Employing a rather simple experimental design, Bonnu et al. performed a miRNA analysis from two tissue samples of PCa and two of BPH patients in Indonesia. Of six significantly differentiated miRNAs, the best diagnostic accuracy showed miR-106b-5p, which was subsequently validated within urine exosomes. Although this article does not provide a detailed study design, the results suggest miR-106b-5p as a potential candidate diagnostic biomarker for PCa [52].

Wani et al. focused on the analysis of exosomal miR-2909 and miR-615-3p in a set of patients with PCa, BPH, and bladder cancer patients. While their findings showed a markedly increased amount of miR-615-3p in the exosomes derived from both bladder or prostate cancer urinary samples compared to healthy or BPH patient specimens, miR-2909 was found exclusively in the exosomes of prostate cancer patients. Although in vitro experiments showed its high expression in bladder cancer cells, miR-2909 was absent in the exosomes derived from these cells. In addition to its specificity to PCa, miR-2909 showed a significant correlation with clinicopathological parameters, and it could differentiate patients into three PCa risk groups [53].

Matsuzaki et al. performed a miRNA analysis in EVs extracted by differential centrifugation from whole urine obtained after DRE. miRNAs were detected using a hybridization microarray in a cohort counting 10 PCa patients and four subjects with negative biopsy. Overall, 19 miRNAs showed a significantly higher level in PCa patients (fold change > 1.5, *p* < 0.05). Furthermore, two candidate miRNAs, miR-30b-3p and miR-126-3p, confirmed their discrimination power in the independent cohort by outperforming serum PSA [54].

The urine supernatant of six PCa patients and three healthy controls was used for the NGS profiling experiments conducted by Li et al. Detected miRNAs were validated in a cohort of 47 PCa patients, 29 BPH patients and 25 age- and gender-matched healthy donors. Exosomes were precipitated and isolated using ExoQuick-TC. The sequencing revealed 53 significantly dysregulated miRNA levels between the PCa patients and healthy controls. To confirm the NGS results, the differences in the expression levels of selected miRNAs (miR-375, miR-451a, miR-486-3p and miR-486-5p) were validated by RT-qPCR reactions in the training cohort. The four-miRNA-based classifier was performed with 91% sensitivity and 89% specificity (AUC = 0.979) to distinguish PCa patients and healthy donors. Moreover, miR-375 separated localized vs. metastatic PCa samples, and in combination with miR-451a, it could differentiate PCa from BPH patients [55].

A multi-marker approach is used within the Sentinel platform, which consists of three different tests that are based on the exosomal short non-coding RNAs (sncRNAs) isolated from the urine supernatant. The platform is based on a classification algorithm that reflects the sncRNA expression signature of the tested individual. The discovery phase (PCa and CS test) involved 235 participants and was performed using Affymetrix miR 4.0 arrays. Training and validation included a case-control sample of 1436 subjects, and the validation was performed using the OpenArray platform. Sentinel PCa test based on 85 miRNA and 60 small nucleolar RNA (snoRNA) targets could distinguish between PCa patients and PCa-free individuals with 94% sensitivity and 92% specificity. The Sentinel HG test (122 miRNAs and 25 snoRNAs) is aimed at men diagnosed with PCa to differentiate between lower (GG1-2) and higher-grade disease (GG3-5) with 94% sensitivity and 96% specificity. The Sentinel CS test (130 miRNAs and 66 snoRNAs) can be used to monitor the patient over longer periods, as it can classify low-grade (GG1) and intermediate- and high-grade cancer (GG2-5) with 93% sensitivity and 90% specificity [56].

Due to the distinct methodologies used for EVs isolation and miRNA detection [49,50,51,52,53,54,55,56] and often rather low numbers of patients [52,54,55], there is a small match between molecules detected across listed studies. Only miR-375 was shown to be dysregulated in urinary EVs of PCa patients compared to controls in more than one discovery study [35,55] and independent validation using an alternative detection method [57,58].

## 5. New Frontiers in Urinary miRNA-Based PCa Detection

As suggested before, miRNA analysis in urine suffers from shortcomings that are mainly connected to procedures within the preanalytical phase, e.g., urine collection, sampling, preservation, or storage. Here, the solution should be not only to optimize the preanalytical process, but also to recognize all biological and technological factors that affect miRNA analysis in urine and include them in data evaluation. The second important cause of inconsistency among studies is the detection itself, as every analytical method has its own limitations. In order to eliminate these limitations, new alternative detection methods or approaches could be implemented.

### 5.1. Factors Influencing Urinary miRNA Analysis

One of the important factors not studied often within urine miRNA analysis is the effect of anti-cancer treatment including surgical procedures. Konoshenko et al. evaluated the effect of radical prostatectomy on the level of diagnostic miRNAs identified in their previous research [36]. All 19 miRNA ratios examined were altered in at least one biological material (urine supernatant, urine exosomes, blood plasma) analyzed when comparing the miRNA levels before and after radical prostatectomy [59]. Such changes can be explained not only by tumor tissue resection leading to a decrease in oncomirs, but also by an increased rate of healing or regeneration, causing an increase in tumor suppressor miRNAs. Similarly, miRNA level can be altered as a result of drug administration [60] and has to be assumed mainly when analyzing post-operative miRNA levels in urine.

Another major factor that needs to be addressed is the intraindividual variability of miRNA levels in urine. While interindividual variability displays how differently a clinical biomarker is expressed between compared groups, intraindividual variability shows changes in the levels within one subject across repeated measurements. Therefore, a reliable biomarker should show low intraindividual variability and stability over time. This parameter is studied within the scope of protein biomarkers [61], but its relation to circulating miRNAs has only recently been mentioned [62]. In one of the few studies, Jeon et al. examined the longitudinal stability of selected miRNAs in serial urine samples from patients with prostate cancer. In general, urine miRNA profiles showed lower intraindividual variability (ρ = 0.67 ± 0.10) than variability between patients (ρ = 0.40 ± 0.15). Serial samples from one patient differed more in the number of detected miRNAs but not in their abundance. This suggests that variability is caused by rather analytical than biological factors. Overall, this study demonstrates the importance of assessing intraindividual variability, which could be a key exclusion factor within biomarker identification [63].

Since hematuria frequently occurs during prostate cancer [64], hemolysis seems to have a major impact on the presence and quantity of miRNA in biofluids. The content released from red blood cells (RBC) can cause a significant variation in miRNA levels, leading to the discovery of biomarkers not specific to the disease but to the sample condition [65]. There is a large number of miRNAs known to be enriched in RBC, such as miR-451, which was already proposed for the sensitive detection of hemolysis [66]. In addition, Kirschner et al. presented globally increased miRNA levels with a number of specifically dysregulated miRNAs in hemolyzed samples. Among the molecules most affected by hemolysis that can be found are miR-16, miR-17, miR-92a, miR-106a, and miR-210 [65]. miR-16 was proposed as a biomarker candidate in the diagnosis of several diseases, including cancer [67]. This miRNA is widely used as a reference gene for normalization of qPCR data [68]. Moreover, urinary miR-16 [41], miR-92a [28,36], and miR-451 [28] have been identified as possible diagnostic markers for PCa. Therefore, it is at least misleading to consider these molecules as tumor-specific biomarkers without the knowledge of other biological and technical contexts.

Urinary EVs represent a remarkable source of potential biomarkers since they reflect the molecular nature of the parental cells [69]. Moreover, they are non-invasive and available in larger volumes, but their use for cancer detection, progression, or treatment poses challenges as well. Pre-analytical variables are paramount to the quality and quantity of obtained uEVs and should be carefully considered in the study design. Although standard operating procedures are not established in EV research, the protocols employed should be reported according to the Minimal Information for Studies of Extracellular Vesicles 2018 (MISEV2018) guidelines [70]. Most of the aforementioned studies did not provide detailed information on urine collection, its processing, quality control, and storage conditions, which are important factors when data variability and reproducibility are concerned. The pre-analytical protocol of one study included digital rectal examination as a means to increase the levels of prostate-specific EVs [71].

The isolation methods for EVs vary among the studies, but precipitation-based techniques are used most often. The choice of separation technique may influence the characteristics of EVs, their analysis, and the presence of co-isolates such as uromodulin [72,73]. Polymerization of this most abundant urinary protein can cause entrapment of EVs during centrifugation, leading to their loss [74]; therefore, its elimination needs to be clearly addressed in the study design. All separation methods acquire only a subset of EVs that may not contain the target miRNA, but they might also enrich a subset of Evs of interest. Since urine production can be highly variable, the concentration of a candidate miRNA should be normalized to an absolute or a relative excretion rate of uEVs [70]. The description of the normalization method is often missing in the cited studies. Furthermore, many of the detected miRNAs were not validated in an independent cohort or verified in more than one laboratory. Factors affecting urinary miRNA levels within prostate cancer diagnosis are referred to in Table 3.

### 5.2. Alternative Methods of Detection

At present, the most common techniques for miRNA quantification are qPCR-based methods. Although these method are the gold standard, they have several disadvantages that can make miRNA detection quite difficult or inaccurate. In general, inconsistencies across the literature are not observed within the technical design, but rather within data processing and analysis, because no consensus on the process has been adopted. To improve this, a coherent and sequential procedure for data evaluation could be useful. From this point of view, Bryzgunova et al. presented a new perspective on PCa classification using urine miRNAs. Based on their extensive research in this field, they proposed a four-block data analysis algorithm that can be described as a series of steps that allow for the classification of suspected prostate cancer patients. The expression data of selected urine miRNAs and their ratios are analyzed according to pre-defined arbitrary cut-off ranges, which should compensate for the heterogeneity of the miRNA expression but also of the group population. Subjects are classified into these four steps as “Prostate disease”, “PCa”, “Not PCa”, or “Healthy” with a total accuracy of 97.5% [75]. Often mentioned new approaches in data processing are machine learning methods [35,76]. In addition to conventional analyses of small RNA sequencing data, Markert et al. used machine learning algorithms to identify a panel of 22 miRNAs that allowed them to distinguish between PCa patients and negative controls [76].

In order to achieve the detection of miRNA in urine at a lower concentration or to simplify the analysis, several alternative approaches have been proposed over the last five years. The main advantage of these methods is the direct detection of RNA without the need for reverse transcription and further amplification. In their study, Lee et al. focused on miRNAs in urine exosomes, which represent a promising source of cancer-related markers but are, however, isolated in laborious and multi-step procedures. To simplify the process, they demonstrated in situ detection using bi-labeled hairpins (molecular beacons) that include complementary sequences to miR-375 and miR-574-3p, which are both proposed markers of prostate cancer. Given their nano-size, the molecular beacons are able to penetrate exosomes where they hybridize with the miRNA strand and produce a fluorescent signal. Although this detection system holds considerable potential, the question of how urine affects individual parts of molecular beacons needs to be answered [57]. Similarly, Saha et al. presented a two-step competitive hybridization assay that converts microRNA concentrations to electrochemical signals. Within the first step, the unlabeled target miRNAs of the biological sample are captured on probes immobilized on the transducer. The second step involves hybridization of the unreacted capture probes with the signaling DNA barcodes. Due to the inversely proportional signal (les target miRNAs mean more unreacted probes and thus a higher electrochemical signal), this method is capable of detecting extremely low concentrations of selected biomolecules, as shown in the successful analysis of vesicular miR-200b in urine from prostate cancer patients [77]. To overcome reverse-transcription PCR limitations, Kim et al. developed a hydrogel-based hybridization chain reaction (HCR). This method involves the targeted hybridization of urinary exosomal miRNAs and a DNA oligomer as a miRNA probe anchored within the hydrogel. The reaction is then initiated with the sequential binding of the biotinylated universal adapter, neutravidin, and biotinylated universal initiator. Such a complex undergoes hybridization with biotinylated universal hairpins followed by chain reaction amplification of the hairpin set. Finally, the fluorescent reporter binds to the biotinylated sites and leads to the signal. Using this technique and ratiometric analysis, they subsequently detected a statistically higher ratio of miR-6090 to miR-3665 in prostate cancer patients than in healthy controls, suggesting this method as a new diagnostic platform for a non-invasive liquid biopsy analysis [78].

Using electrochemistry, Kim et al. performed the detection of miRNAs in urine from PCa patients with a graphene-based electrical sensor connected to a field-effect transistor. Peptide nucleic acids within this sensor chip hybridize with miRNAs in a biological sample and mediate a change in electrical signal. The tested molecules miR-21, miR-1246, and let-7b showed a significantly higher level in patients with prostate cancer compared to patients without the disease. Due to the label-free detection, good durability, and the dynamic range, this device could serve for rapid diagnosis based on urine miRNA assessment [79].

Another new approach to analyze EV-associated miRNAs in urine is to use nanotechnology. One of the proposed systems employs an electrostatic collection of EVs based on the interaction of the negatively charged surface of urine EVs (pH 6–8) and positively charged nanowires. After in situ extraction, EV-encapsulated miRNAs can be analyzed using standard high-capacity screening methods, as in the case of Yasui et al., who performed a hybridization miRNA microarray and unveiled a large number of miRNAs dysregulated in PCa samples in comparison with non-cancer samples [80]. Nucleic acids can also be used to guide so-called self-assembly nanoparticles. NA nanostructures can be later analyzed using numerous biophysical methods such as electrochemical, optical, advanced microscopy, or spectroscopy. Correspondingly, Li et al. designed miRNA-driven self-assembly of Au nanospheres for PCa diagnosis. In this system, an endogenous miR-107 sequence is recognized by target nanospheres, thus triggering the self-assembly of plasmonic nanostructures detectable by surface-enhanced Raman scattering. When evaluated in clinical samples, the spectral signal unveiled a significantly higher level of miR-107 (*p* < 0.05) in the urine samples from PCa patients when compared to healthy controls. Moreover, nanostructures allowed for miRNA quantification without any pre-processing step, as it would be required for instance in PCR-based methods [81].

Sometimes, the pathophysiology of the tumor is so complex and heterogenous that one class of biomarkers does not sufficiently display various aspects of the systemic response to a present tumor. For this reason, efforts have been made to develop multi-marker systems. Davey et al. proposed a panel that combined several mRNA and miRNA targets isolated from urinary EVs. To simplify the analysis, they utilized Vn96 peptide-mediated EV capture technology followed by qPCR-based detection of selected markers. The panel consisting of FOLH1, HPN, CD24, TMPRSS2-ERG, ITSN1, ANXA3, SLC45A3, miR-375, and miR-574 distinguished prostate cancer from benign states with an AUC of 0.843. The combination with PCA3 and clinical–pathological data improved the accuracy of the multi-marker system to an AUC of 0.955 [58]. New alternative approaches in urinary miRNAs detection are listed in Table 4.

## 6. Conclusions

Despite a large amount of experimental data, published results need to be independently validated so that urinary microRNAs can be implemented as standard clinical biomarkers for prostate cancer diagnostics. The main reason is inconsistencies across studies, whether they differ in sample manipulation, the technical aspect of analysis, or subsequent data evaluation. One way to refine the analysis of miRNAs in urine is to focus on the molecules encapsulated within extracellular vesicles, because this fraction represents a discrete department of which the content is not easily affected by the urine environment. However, the analysis of EVs has several shortcomings mainly related to vesicle isolation, which, depending on the methodology used, achieves various efficiencies. For each urine fraction, qPCR remains the standard method for miRNA analysis, even if additional steps (reverse transcription, etc.) are necessary and data evaluation is dependent on correct normalization. The solution could be provided by new methods based on different physical–chemical principles (electrochemistry, in situ fluorescence, and spectroscopy of nanostructures conjugated with miRNAs), which enable quick miRNA assessment in biofluid without previous manipulation with the sample. Despite their considerable potential, these methods need to be properly validated. Another approach is represented by multi-marker systems that combine biomarkers with different biological behavior. However, no method provides accurate results if it is performed on biological material of poor quality. Therefore, special attention should be paid to the pre-analytical phase, which plays a key role in the reproducibility of the results. Only a strict adherence to the widely accepted principles of miRNA analysis guiding pre-analytical steps, but also study design, and the analysis itself, can ensure production of the reliable data.

## Figures and Tables

**Table 1:** Urinary cell-free miRNAs and miRNAs in urine sediment identified as potential biomarkers for prostate cancer detection in the context of study design.

Author, Year	Urine Fraction	Screening (Method/Samples)	Validation (Method/Samples)	Proposed Biomarkers/Comments	Reference
Byun, 2021	urine supernatant	Agilent Human miRNA Microarray/14 PCa, 5 BPH	qPCR/ cohort 1: 9 PCa, 8 BPH; cohort 2: 44 PCa, 39 BPH	↑ miR-1913 to miR-3659 ratio	[32]
Fredsøe, 2018	urine supernatant	RT-qPCR array/188 PCa, 20 BPH	RT-qPCR array/197 PCa, 20 BPH	↑ miR-222-3p, miR-24-3p, miR-30c-5p/diagnostic model	[33]
Fredsøe, 2019	urine supernatant	RT-qPCR array/404 PCa, 42 BPH; merged cohorts from previous study	RT-qPCR array/cohort 1: 214 PCa, 99 BPH; cohort 2: 139 PCa, 148 BPH	↑ miR-222-3p, miR-24-3p, miR-30c-5p/diagnostic model	[34]
Lekchnov, 2018	urine supernatant, urine Evs	RT-qPCR array/10 PCa, 10 HC, 10 BPH	-	supernatant: ↑ miR-107-miR-26b-5p, ↑ miR-375-3p-miR-26b-5p; Evs: miR-20a-5p-miR-16-5p, miR-30b-5p-miR-16-5p, miR-31-5p-miR-16-5p, miR-24-3p-miR-200b-3p/miRNA pairs	[35]
Konoshenko, 2020	urine supernatant, urine Evs	based on previous study [35], RT-qPCR array	qPCR/10 PCa, 11 HC, 8 BPH	↑ miR-125b-miR-30e, ↑ miR-200-miR-30e, ↑ miR-205-miR-30e, ↑ miR-31-miR-30e, ↑ miR-660-miR-30e, ↑ miR-19b-miR-92a/miRNA ratios	[36]
Hasanoğlu, 2021	urine sediment	Affymetrix GeneChip miRNA 4.0 Arrays/8 PCa, 30 HC	qPCR/8 PCa, 30 HC	↑ miR-320a	[37]
Guelfi, 2018	urine sediment/ exfoliated cells	small RNA sequencing/11 PCa, 11 HC	qPCR/11 PCa, 11 HC	↓ let-7 family	[38]
Ghorbanmehr, 2019	whole urine	-	qPCR/23 PCa, 22 BPH, 20 HC	↑ miR-21-5p, ↑ miR-141-3p, ↑ miR-205-5p	[39]
Nayak, 2020	urine sediment	-	qPCR/33 PCa, 30 HC	↑ miR-182, ↓ miR-187/only in tissue	[40]
Borkowetz, 2020	urine sediment	-	qPCR/50 suspected PCa (26 PCa, 24 tumor-free)	↓ miR-16, ↓ miR-195	[41]
Foj, 2017	urinary sediment, urinary Evs	-	qPCR/60 PCa, 10 HC	Sediment: ↑ miR-21, ↑ miR-375, ↑ miR-141, ↓ miR-214; Evs: ↑ miR-21, ↑ miR-375, ↑ let-7c	[42]

PCa—prostate cancer, HC—healthy controls, BPH—benign prostatic hyperplasia, ↑—upregulated in PCa, ↓—downregulated in PCa.

**Table 2:** Exosomal miRNAs identified as potential biomarkers for prostate cancer detection in the context of study design.

Author, Year	EVs Isolation Method	Screening (Method/Samples)	Validation (Method/Samples)	Proposed Biomarkers	Reference
Xu, 2017	hydrostatic filtration dialysis, ultracentrifugation	qPCR/60 PCa, 37 BPH, 24 HC	-	↑ miR-145-5p	[49]
Ku, 2021	automated acoustic trapping	NGS/46 PCa GG ≥ 4, 127 PCa GG ≤ 3 + Bx-negative samples	In silico, TCGA prostate dataset/497 subjects	↓ miR-1, ↑ miR-23b, ↑ miR-27a	[50]
Danarto, 2020	Exiqon miRCURY	qPCR/60 PCa, 20 BPH	-	↑ miR-21-5p, ↓ miR-200c-3p	[51]
Bonnu, 2021	QIAGEN exosomal Kit	NanoString nCounter Expression Assay/2 PCa, 2 BPH—tissue samples	qPCR/10 PCa, 10 BPH	↑ has-mir-106b-5p	[52]
Wani, 2017	Exiqon miRCURY	qPCR/90 PCa, 10 BPH, 60 BCa, 50 HC	-	↑ miR-2909, ↑ miR-615-3p	[53]
Matsuzaki, 2021	differential centrifugation	Affymetrix miRNA microarray 2.0/10 PCa, 4 HC	qPCR/28 PCa, 25 HC	↑ miR-30b-3p, ↑ miR-126-3p	[54]
Li, 2021	ExoQuick-TC	small RNA sequencing/6 PCa, 3 HC	qPCR/47 PCa, 29 BPH, 25 HC	↓ miR-375, ↑ miR-451a, ↑ miR-486-3p, ↑ miR-486-5p	[55]
Wang, 2020	Exosome RNA Isolation Kit (Norgen Biotek)	Affymetrix GeneChip miRNA 4.0 Arrays/146 PCa, 89 HC	qPCR OpenArray/868 PCa, 568 HC	Sentinel PCa, Sentinel CS and Sentinel HG	[56]

EVs—extracellular vesicles, GG—grade group, BCa—Bladder cancer, ↑—upregulated in PCa, ↓—downregulated in PCa.

**Table 3:** Recorded factors and their effect on urinary miRNA analysis.

Factor	Effect/Consequence	Significant miRNAs/Comments	Reference
anti-cancer treatment (radical prostatectomy)	miRNA level alteration	miR-19b, miR-30e, miR-31, miR-125b, miR-200b, miR-205, miR-375, miR-378, miR-425, miR-660	[59]
intraindividual variability	changes in level within one subject across repeated measurements	miR-3195, let-7b-5p, miR-144-3p, miR-451a, miR-148a-3p, miR-512-5p, miR-431-5p/intrastable miRNAs	[60]
hemolysis	variation in miRNAs enriched in RBC	miR-16, miR-17, miR-92a, miR-106a, miR-210, miR-451	[65,66]
inappropriate reference gene	unreliable data normalization	miR-16	[68]
EV separation method	enrichment of different EV subpopulations and content	-	[72]
presence of non-EV components	decrease in EV yield and change in levels of miRNA	miR-21, miR-375 and miR-204	[74]

RBC—red blood cells.

**Table 4:** Alternative approaches or methods for urinary miRNA detection within prostate cancer diagnosis.

Author, Year	Method/Approach	Advantage	Disadvantage	Reference
Bryzgunova, 2019	qPCR data evaluation using four-block data analysis algorithm	simplification of miRNA expression, analysis in more urine fractions, compensation of heterogeneity	algorithm based on the analysis of a smaller group of patients, disadvantages connected to qPCR method	[75]
Markert, 2021	machine learning classification algorithm for data analysis	low dependence on the (error-free) measurability of a single marker	algorithm based on the analysis of a small sample size	[76]
Lee, 2018	bi-labeled molecular beacons	direct detection	unknown effect of urine on technology, suitable exosomes isolation	[57]
Saha, 2021	two-step competitive hybridization assay	direct detection, high sensitivity	one marker per analysis, signal normalization	[77]
Kim, 2021	hydrogel-based hybridization chain reaction	analysis without target amplification, low urine volume, ratiometric analysis	instrumentation, needs to be validated on extended cohorts	[78]
Kim, 2021	graphene-based electrical sensor	label-free detection, durability, dynamic range	instrumentation, limited number of measured biomarkers	[79]
Yasui, 2017	electrostatic collection of EVs + standard screening methods	standardized, high efficiency EV collection, small urine volume (1 mL)	only improving EVs extraction, disadvantages connected to subsequent method	[80]
Li, 2019	detection of miRNA-driven self-assembly nanospheres	quantification without pre-processing step, high sensitivity and specificity	synthesis of nanospheres, instrumentation	[81]
Davey, 2020	multi-marker system	detection in EVs, unified peptide-mediated EV capture, combination of different types of markers	disadvantages connected to qPCR method	[58]

EV—extracellular vesicle.
